# Kidney Development in the Absence of *Gdnf* and *Spry1* Requires *Fgf10*

**DOI:** 10.1371/journal.pgen.1000809

**Published:** 2010-01-15

**Authors:** Odyssé Michos, Cristina Cebrian, Deborah Hyink, Uta Grieshammer, Linda Williams, Vivette D'Agati, Jonathan D. Licht, Gail R. Martin, Frank Costantini

**Affiliations:** 1 Department of Genetics and Development, Columbia University, New York, New York, United States of America; 2 Department of Medicine, Mount Sinai School of Medicine, New York, New York, United States of America; 3 Department of Anatomy, University of California San Francisco, San Francisco, California, United States of America; 4 Northwestern University Feinberg School of Medicine, Department of Medicine, Chicago, Illinois, United States of America; Harvard Medical School, United States of America

## Abstract

GDNF signaling through the Ret receptor tyrosine kinase (RTK) is required for ureteric bud (UB) branching morphogenesis during kidney development in mice and humans. Furthermore, many other mutant genes that cause renal agenesis exert their effects via the GDNF/RET pathway. Therefore, RET signaling is believed to play a central role in renal organogenesis. Here, we re-examine the extent to which the functions of *Gdnf* and *Ret* are unique, by seeking conditions in which a kidney can develop in their absence. We find that in the absence of the negative regulator *Spry1*, *Gdnf*, and *Ret* are no longer required for extensive kidney development. *Gdnf−/−;Spry1−/−* or *Ret−/−;Spry1−/−* double mutants develop large kidneys with normal ureters, highly branched collecting ducts, extensive nephrogenesis, and normal histoarchitecture. However, despite extensive branching, the UB displays alterations in branch spacing, angle, and frequency. UB branching in the absence of *Gdnf* and *Spry1* requires *Fgf10* (which normally plays a minor role), as removal of even one copy of *Fgf10* in *Gdnf−/−;Spry1−/−* mutants causes a complete failure of ureter and kidney development. In contrast to *Gdnf* or *Ret* mutations, renal agenesis caused by concomitant lack of the transcription factors ETV4 and ETV5 is not rescued by removing *Spry1*, consistent with their role downstream of both RET and FGFRs. This shows that, for many aspects of renal development, the balance between positive signaling by RTKs and negative regulation of this signaling by SPRY1 is more critical than the specific role of GDNF. Other signals, including FGF10, can perform many of the functions of GDNF, when SPRY1 is absent. But GDNF/RET signaling has an apparently unique function in determining normal branching pattern. In contrast to GDNF or FGF10, *Etv4* and *Etv5* represent a critical node in the RTK signaling network that cannot by bypassed by reducing the negative regulation of upstream signals.

## Author Summary

Kidney development requires the secreted protein GDNF, which signals via its cellular receptor RET to promote growth and branching of the ureteric bud, the progenitor of the collecting duct system. The transcription factors ETV4 and ETV5 regulate gene expression in response to GDNF. We report that deleting *Spry1*, a feedback inhibitor downstream of RET, largely rescues kidney development in mice lacking GDNF or RET, although not in those lacking ETV4 and ETV5. Thus, GDNF and RET become dispensable in the absence of SPRY1, when their roles can be largely assumed by other signals and receptors, while ETV4 and ETV5 remain indispensible. We identify FGF10 as the signal responsible for kidney development in the combined absence of GDNF/RET signaling and SPRY1 negative regulation. But while the ureteric bud branches extensively in *Gdnf−/−;Spry1−/−* and *Ret−/−;Spry1−/−* kidneys, its pattern of branching is severely perturbed. This points to a unique function of GDNF in ureteric bud patterning.

## Introduction

Signaling by the secreted protein GDNF through the RET receptor tyrosine kinase (RTK) and the GFRα1 co-receptor plays a central role in the initiating event of kidney development, the outgrowth of the ureteric bud (UB) from the Wolffian duct (WD) into the metanephric mesenchyme (MM). They are also important for the subsequent growth and branching of the UB to form the renal collecting duct system. This is apparent not only from the lack of UB development in *Gdnf, Ret*, and *Gfra1* mutants in mice [1]–[5] and humans [6], but also from the observation that most of the other genes whose absence causes renal agenesis are upstream regulators of *Gdnf* or *Ret* expression [7]. We have recently reported that expression in the UB of the ETS transcription factors ETV4 and ETV5 is upregulated by GDNF/RET signaling, and that *Etv4−/−;Etv5−/−* double homozygous mice fail to develop kidneys. Thus, the effects of GDNF/RET signaling on UB branching morphogenesis are largely transduced via ETV4 and ETV5 [8].

The mechanism by which GDNF/RET signaling induces epithelial branching remains to be fully elucidated. In the WD, it initially promotes cell movements that precede and lead to the formation of the UB [9], and it then induces UB outgrowth from the duct [10],[11]. In the UB tips, it increases cell proliferation [12],[13], a likely prerequisite for branching. Furthermore, because GDNF is capable of acting as a chemoattractant for cultured kidney cells [14],[15], it has been suggested that GDNF may act as a chemoattractant for UB tips *in vivo*, thereby promoting and patterning their branching [10],[11],[16].

Here, we have further investigated the role of GDNF/RET signaling by identifying conditions under which the kidney can develop in the absence of either GDNF or RET. To achieve this, we employed a null allele of Sprouty1 (*Spry1*), a negative feedback inhibitor of RTK signaling, which modulates the response to GDNF during kidney development. *Spry1−/−* mutants show a pervasive defect in the development of the ureteric tree, including formation of supernumerary buds from the WD, which develop into multiplex ureters and kidneys, and an increase in the number and diameter of UB branches in the developing kidneys [17],[18]. The molecular mechanism of Sprouty protein function is incompletely understood. Engineered expression of Sprouty in cells leads to inhibition of signaling through the MAP kinase (MAPK) pathway, but effects have also been observed on the PI3K and PLCγ signaling pathways downstream of RTKs [19],[20].

It was previously found that removing one *Spry1* allele corrected the renal hypoplasia in *Gdnf*+/− heterozygous mice, and that removing one *Gdnf* allele corrected the abnormal UB branching in *Spry1−/−* mice. These findings demonstrated that the balance between GDNF and SPRY1 levels is critical for normal kidney development [17],[18]. We have now further tested this idea by examining the consequences of eliminating *Gdnf* and *Spry1* (or all *Ret* and *Spry1*). Surprisingly, such doubly homozygous mutant mice developed two large and well-formed kidneys, each with a single, normally-positioned ureter. Thus, in the absence of GDNF/RET signaling, other factors must be able to support normal UB outgrowth and extensive UB branching, but only when SPRY1 is absent. We provide *in vivo*, genetic evidence that FGF10 is one such factor, consistent with the previous observation that exogenous FGFs are capable of inducing budding by the Wolffian duct in organ culture [21]. However, our data also reveal that the specific pattern of UB branching is abnormal in *Ret−/−;Spry1−/−* and *Gdnf−/−;Spry1−/−* double mutant kidneys. Therefore, although endogenous FGF10 and perhaps other factors can promote extensive UB branching, GDNF appears to serve a unique role in the patterning of UB branching morphogenesis. Finally, we show that, unlike the rescue of *Gdnf* or *Ret* mutations, the lack of both *Etv4* and *Etv5* cannot be overcome by removing *Spry1*. Thus, these two transcription factors represent a critical link in a signaling network downstream of Ret and other RTKs.

## Results

### Kidney development in the absence of *Gdnf* and *Spry1*, or *Ret* and *Spry1*

**Figure 1:**
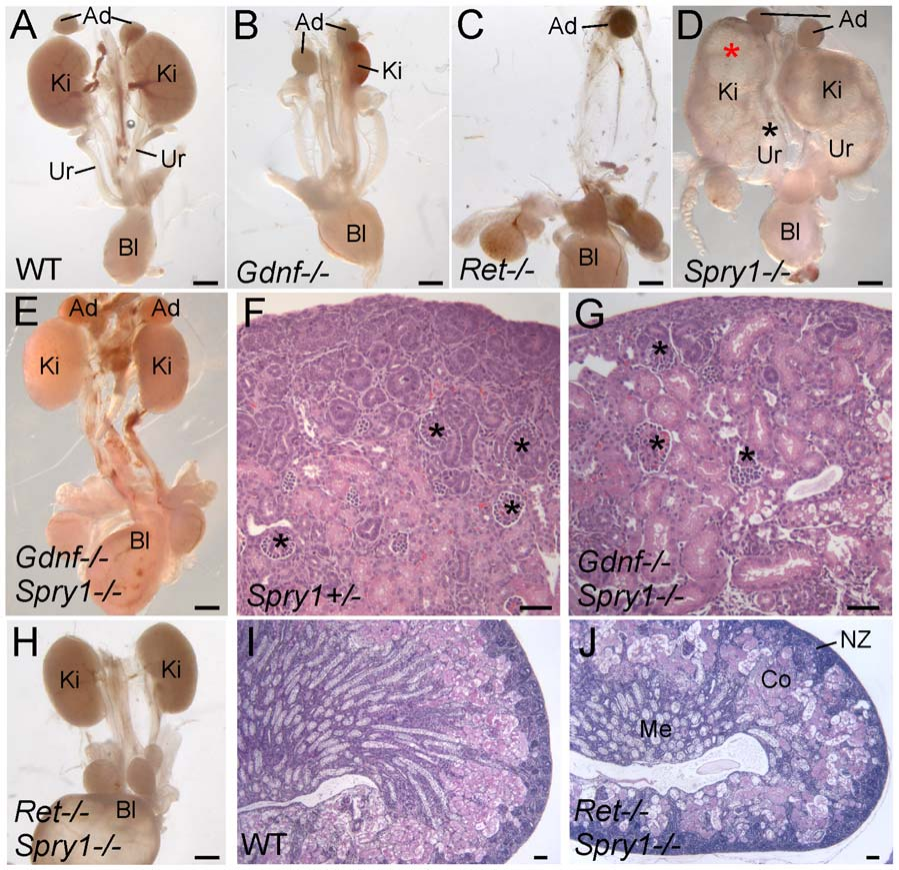
Loss of *Spry1* rescues kidney development in *Gdnf−/−* or *Ret−/−* mice. (A–E,H) excretory systems dissected from newborn mice of the indicated genotypes shown in whole mount. Note that the ureter in the *Spry1−/−* mutant is greatly expanded (black asterisk) and the kidneys are cystic (red asterisk). (F,G) H&E stained sections showing histology of cortex and nephrogenic zone in control (*Spry1*+/−) and *Gdnf−/−*;*Spry1−/−* kidneys, revealing a normal overall organization with well-differentiated glomeruli (*) (I,J) PAS-stained sections of wild-type and *Ret−/−*;*Spry1−/−* double mutant kidneys, showing normal overall organization with renal cortex, medulla, and outer nephrogenic zone. Abbreviations: Ad, adrenal gland; Bl, bladder; Co, cortex; Ki, kidney; Me, medulla, NZ, nephrogenic zone; Ur, ureter. Scale bars 1 mm in A-E and H, 100 µm in F,G,I,J.

*Gdnf−/−* newborn (P0) mice display ∼80% renal agenesis and ∼20% severe renal hypodysplasia [1]–[3] (n = 28) (Figure 1A and 1B) (for statistical purposes, we count each of the two potential kidneys as a separate sample [22]). In contrast, we found that newborn *Gdnf−/−;Spry1−/−* (abbreviated *GGSS*) mice displayed only 11% renal agenesis (n = 18) and 89% of kidneys were normally shaped and only slightly smaller than controls (cross-sectional area 70±11% of wild-type) (Figure 1E). Unlike *Spry1−/−* mice (Figure 1D), *GGSS* newborns never showed hydroureter, although the bladder was often filled with urine (Figure 1E), indicating that the ureters were correctly connected to the bladder, and thus suggesting that the site of outgrowth of the UB from the WD had been normal [23],[24].

**Figure 2:**
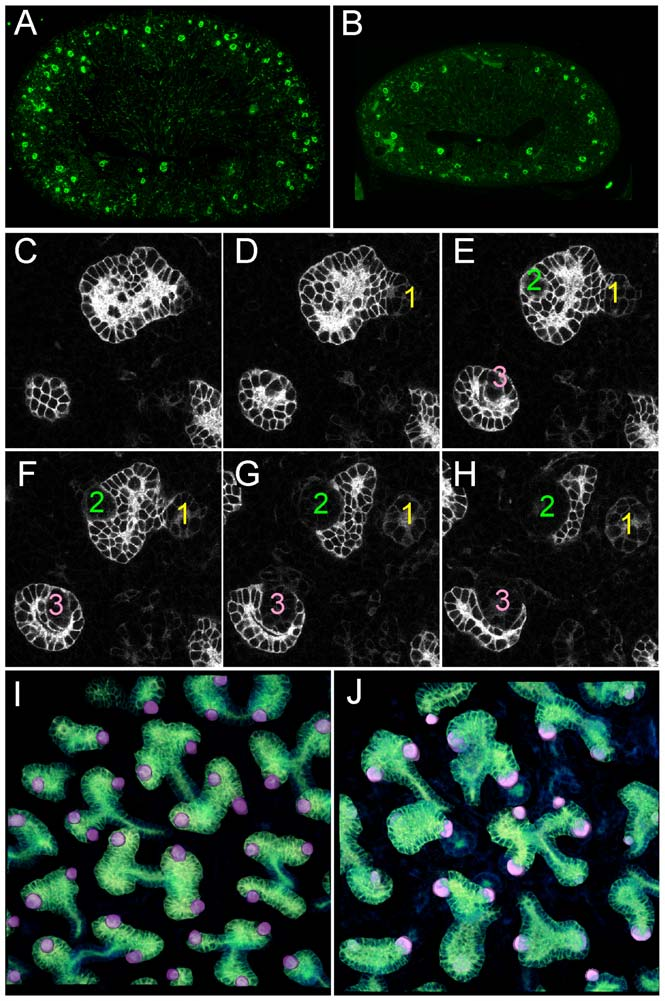
Numerous nephron and normal nephron–UB connections are observed in double mutant kidneys. (A,B) Podocalyxin staining of nascent glomeruli in wild-type (A) and *Ret−/−;Spry1−/−* (B) kidneys at P0, showing numerous, cortically located nephrons in the double mutant, as in the wild-type. (C–H) Six optical sections at different Z-levels of a *Gdnf−/−*;*Spry1−/−* E15.5 kidney carrying *Hoxb7/myrVenus*. The sites where the UB connects to nephrons are visible as “holes” in the myrVenus-labeled UB, as the connecting tubule expresses little or no myrVenus. The connections of three nephrons (1, 2, 3) can be followed at different levels of the image stack. (I) Volume rendering of a wild-type kidney, with nephron connection sites indicated by the pink dots. (J) Volume rendering of double mutant kidneys shown in (C–H), showing normal number and positions of nephron connections per UB tip.

These observations raised the possibility that in the absence of *Gdnf* and *Spry1*, kidney development was supported by another GDNF-family ligand, such as Neurturin, which is expressed in the developing kidney [25]. To investigate this possibility we generated *Ret−/−;Spry−/−* (*RRSS*) newborn mice, as RET is the common signaling receptor for all GDNF family ligands[26]. Whereas *Ret−/−* newborn mice display renal agenesis (∼70%) or severe renal hypodysplasia (∼30%) (Figure 1C), 88% of the *RRSS* double mutants (n = 26) developed fairly large and well-formed kidneys (cross-sectional area 73±15% of wild-type) with apparently normal ureters (Figure 1H). Histological analysis of *GGSS* and *RRSS* kidneys indicated that the overall organization into renal papilla, medulla, cortex, and nephrogenic zone was essentially normal, with well differentiated collecting ducts, nephron epithelia and glomeruli in both double mutant genotypes (Figure 1F, 1G, 1I, and 1J and data not shown). Consistent with these findings, many podocalyxin-positive glomeruli were observed in the cortex of double mutant kidneys, although they were reduced in number (52±6% of wild-type in P0 *RRSS* mutants) (Figure 2A and 2B).

Despite their apparently functional kidneys, *GGSS* and *RRSS* mice did not survive beyond 3–4 days after birth, presumably because removing *Spry1* does not correct the multiple defects in the nervous system caused by lack of *Gdnf* or *Ret* [26].

### UB branching is extensive, but abnormally patterned, in *Gdnf−/−;Spry1−/−* and *Ret−/−;Spry1−/−* kidneys

**Figure 3:**
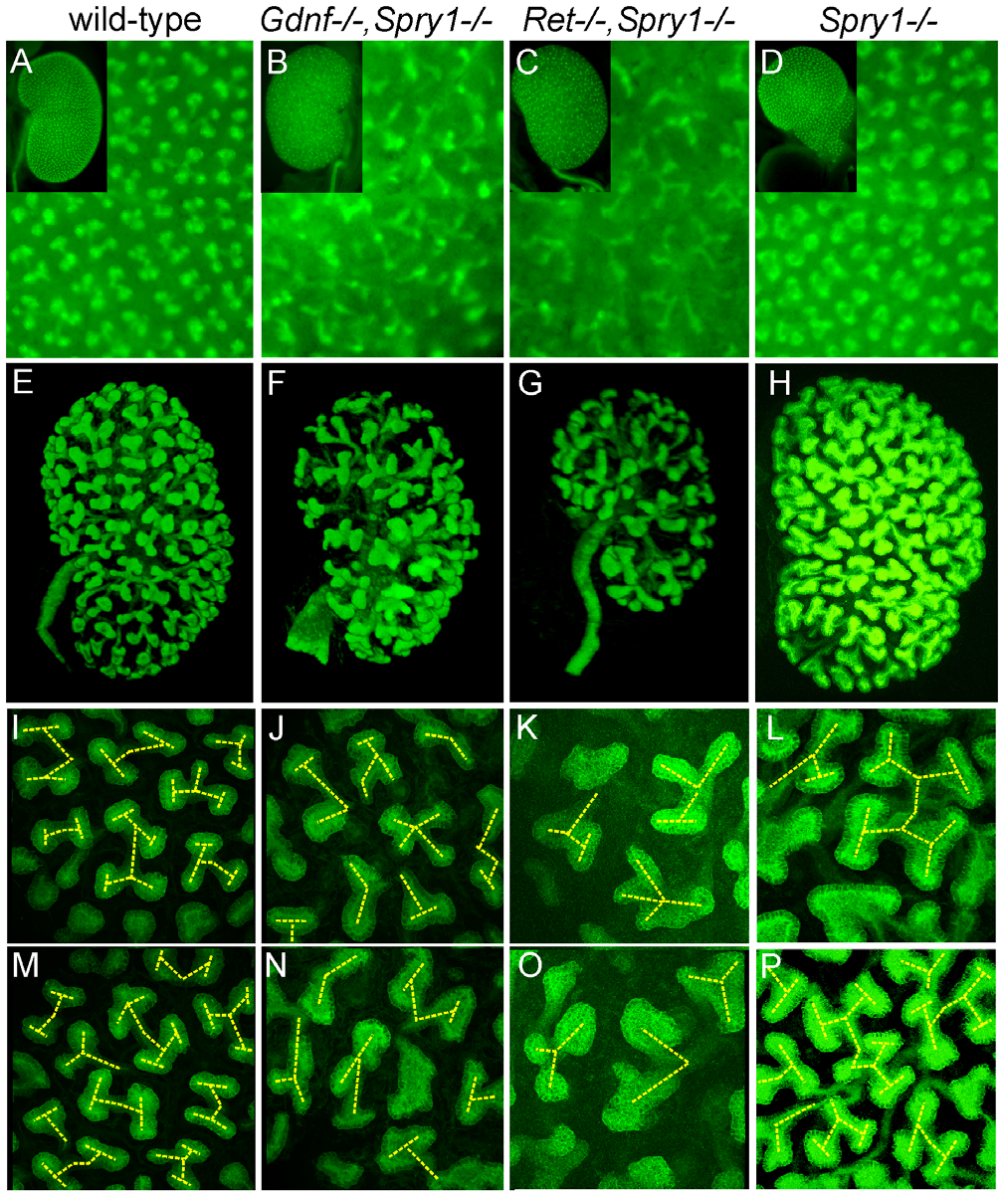
Extensive but irregular UB branching in *Gdnf−/−; Spry1−/−* and *Ret−/−; Spry1−/−* double mutant kidneys. (A–D) Newborn stage kidneys, all carrying the *Hoxb7/myrVenus* transgene to label the UB branches. Each panel shows a high magnification view of the kidney surface, revealing the shape and organization of branching UB tips; insets show the entire kidney in whole mount. Wild-type kidneys (A) have evenly spaced UB tips with a regular branching pattern, whereas *Gdnf−/−*;*Spry1−/−* (B) and *Ret−/−*;*Spry1−/−* (C) double mutant kidneys have highly irregular branching. *Spry1−/−* kidneys (D) have regularly branched, but swollen UB tips. (E–P) 3D volume rendering of E15.5 kidneys. (E–H) Whole kidneys from embryos of the indicated genotypes, carrying *Hoxb7/myrVenus*. (I–P) Higher power views of two representative surface regions of each genotype. The 3D images were generated from confocal Z-stacks, using Volocity (E–H) or ImageJ (I–P). The yellow dashed lines indicate an interpretation of the branching patterns. While most UB branches in the wild-type (I,M) and *Spry1−/−* (L,P) kidneys show a reiterative pattern of terminal bifurcation, with branches forming at right angles to their predecessors, most UB branches in the double mutants (J,K,N,O) fail to conform to this pattern, and instead display a variety of abnormal shapes and branching patterns.

As GDNF/RET signaling is important for UB growth and branching, we crossed into the mutant backgrounds a *Hoxb7/myrVenus* transgene, which fluorescently labels the WD and UB lineage [27], to visualize UB branching *in vivo* or in cultured kidneys. In P0 wild-type kidneys, branching UB tips are numerous and regularly spaced over the kidney surface (Figure 3A). In *Spry1−/−* kidneys, the UB tips are likewise evenly spaced, but abnormally swollen (Figure 3D). In contrast, although there were numerous UB tips on the surface of GGSS and RRSS kidneys, indicating that the UB had branched very extensively even in the absence of *Gdnf* or *Ret*, the tips were irregularly and less densely arrayed, elongated, and abnormally shaped (Figure 3B and 3C). We also examined the kidneys at E15.5, when branching is less complex and the kidneys are small enough to image by confocal microscopy and perform 3D reconstruction (Figure 3E–3P). Volume rendering of the *Hoxb7/myrVenus*-positive UB tree showed that the double mutants had extensively branched, but the spacing and the branching geometry of UB tips was irregular (Figure 3F and 3G). In such samples, the points where UB tips connect to nephrons could be mapped in three dimensions, and were found to be essentially normal in *GGSS* mutants (Figure 2C–2J), indicating that the double mutant UB tips produce the factors necessary to connect to the nephrons. Higher magnification 3D reconstructions revealed a characteristic pattern of UB branching in wild-type kidneys (Figure 3I–3M), where successive branch generations occur at regular intervals, mostly at right angles to the parental branches (yellow dashed lines). In contrast, this regular pattern was rarely observed in *GGSS* or *RRSS* kidneys, where instead the UB tips were highly irregular in shape, orientation, and branching frequency (Figure 3J, 3K, 3N, and 3O). *Spry1−/−* UB tips resembled the wild type, except for an increased tip diameter (Figure 3L and 3P), indicating that the branching abnormalities in *RRSS* and *GGSS* are not due simply to lack of *Spry1*.

**Figure 4:**
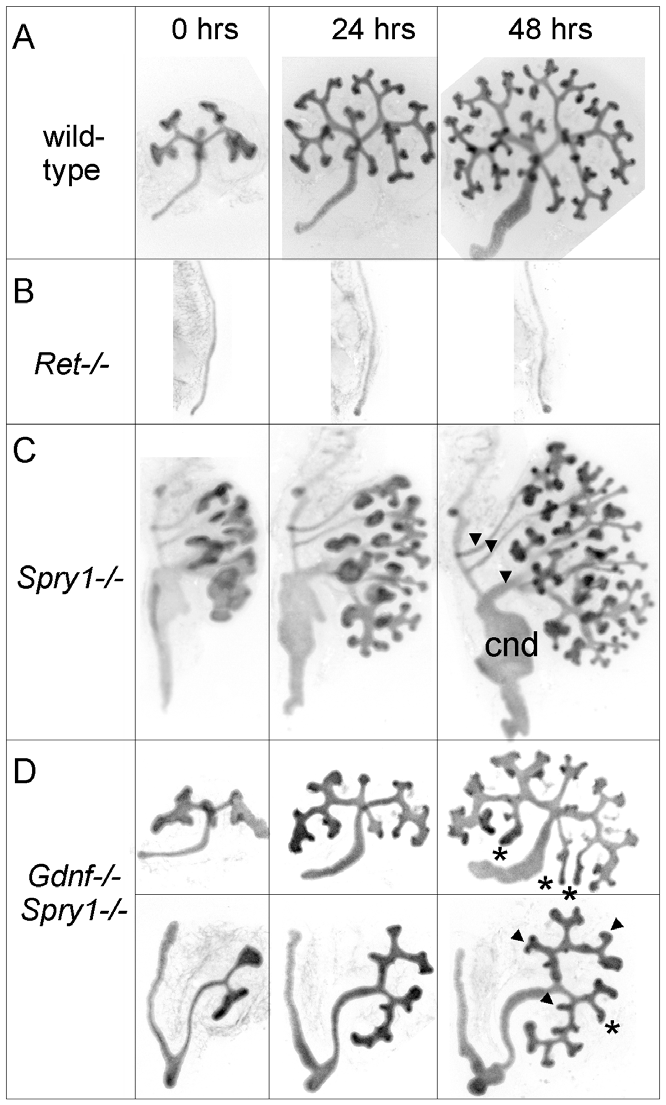
Abnormal branching of double mutant kidneys in organ culture. Kidneys of wild-type (A) and mutant genotypes (B–D), carrying *Hoxb7/myrVenus*, were excised at E12.5, cultured *in vitro*, and photographed at the indicated times. The *Ret−/−* Wolffian duct (B) failed to develop a ureter or kidney, while the *Spry1−/−* kidney (C) has multiple ureters (arrowheads), swollen UB tips and an enlarged common nephric duct (cnd). (D), in two examples of *Gdnf−/−*;*Spry1−/−* mutant kidneys, UB branching is retarded at E12.5, and subsequent branching in culture displays abnormal patterns (asterisks and arrowheads – see text) compared to wild-type.

To examine the initial branching events, we explanted the WD, ureter and kidney at E12.5. Consistent with what was observed in newborn *GGSS* and *RRSS* mutants, the ureter and kidney were nearly always present (88%, n = 26 and 100%, n = 16, respectively). In contrast, few *Gdnf−/−* or *Ret−/−* mutants had ureter and kidney at this stage (20%, n = 30 and 8%, n = 12, respectively). In none of the *GGSS* or *RRSS* mutants were duplicated ureters present, as they are in many *Spry1−/−* mutants. UB branching was somewhat delayed in the *GGSS* and *RRSS* kidneys compared to controls (Figure 4A versus Figure 4D, 0 hours; and data not shown). Several of the wild type, *Spry1−/−*, and *GGSS* E12.5 kidneys were cultured to examine the subsequent branching events. While the *GGSS* kidneys branched extensively in culture, some of the tips elongated abnormally without branching (Figure 4D, asterisks) and some tips grew too slowly (Figure 4D, arrowheads), resulting in an irregularly patterned tree. Thus, while GDNF/RET signaling is not required for the UB to undergo extensive growth and branching when *Spry1* is also absent, it is necessary to impose a regular pattern on UB branching.

### Differentiation of the UB into tip and trunk domains occurs in the absence of *Gdnf/Ret* and *Spry1*

**Figure 5:**
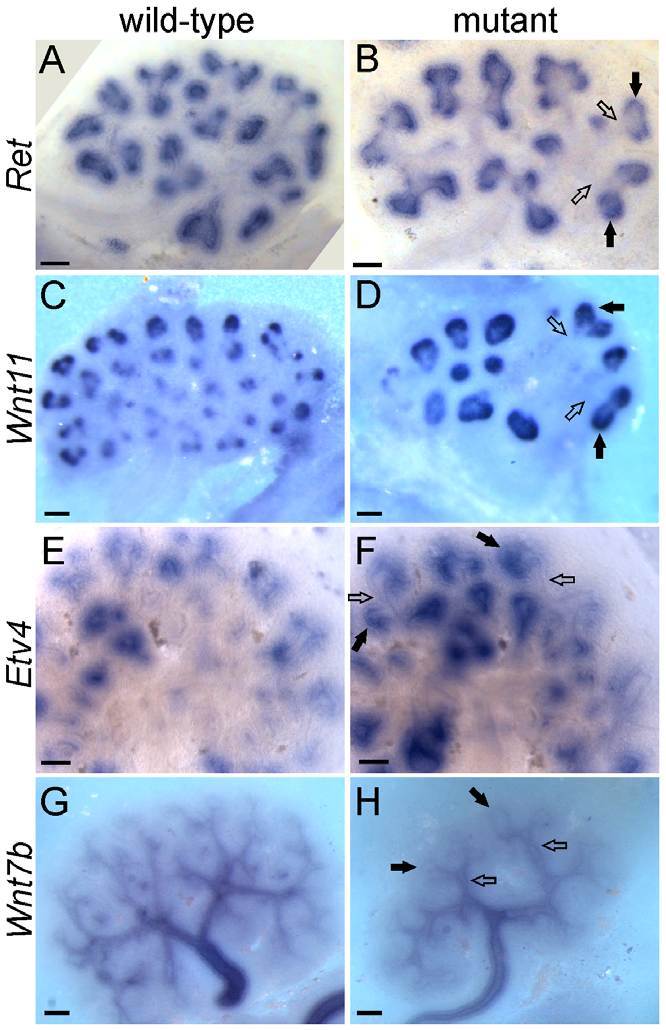
Differential gene expression in tip and trunk domains is retained in *Gdnf−/−;Spry1−/−* and *Ret−/−;Spry1−/−* double mutant kidneys. Whole mount *in situ* hybridization for the UB tip markers *Ret*, *Wnt11* and *Etv4* and the trunk marker *Wnt7b*, in wild-type (A,C,E,G) and double mutant E12.5 kidneys (B,F and H, *Gdnf−/−*;*Spry1−/−*, D, *Ret−/−*;*Spry1−/−*). Solid arrows indicate UB tips and open arrows indicate trunks. Scale bars 100 µm.

The UB tips and trunks maintain different patterns of gene expression throughout kidney development, with many genes expressed specifically in one domain or the other [12],[28],[29]. Many tip-specific genes can be upregulated by exogenous GDNF, and their expression is reduced in a *Ret* hypomorphic mutant [8],[12],[30], suggesting that GDNF/RET signaling may be required to maintain the tip-specific pattern. However, we found that three tip-specific markers, *Ret*, *Wnt11*, and *Etv4*, all of which *normally* require wild-type levels of GDNF/RET signaling for expression in the UB, continued to be expressed in a tip-specific pattern in *GGSS* or *RRSS* double mutants (Figure 5A–5F). The trunk-specific marker *Wnt7b* [31] also retained its normal expression pattern in *GGSS* double mutants (Figure 5G and 5H), indicating that the lack of *Wnt7b* expression in the UB tip does not require GDNF/RET signaling. Therefore, there must be other, *Ret*-independent mechanisms that can establish and maintain tip/trunk differences in gene expression.

### *Fgf10* cooperates with *Gdnf* to promote UB outgrowth and branching morphogenesis, and can largely compensate for the loss of *Gdnf/Ret* in the absence of *Spry1*

**Figure 6:**
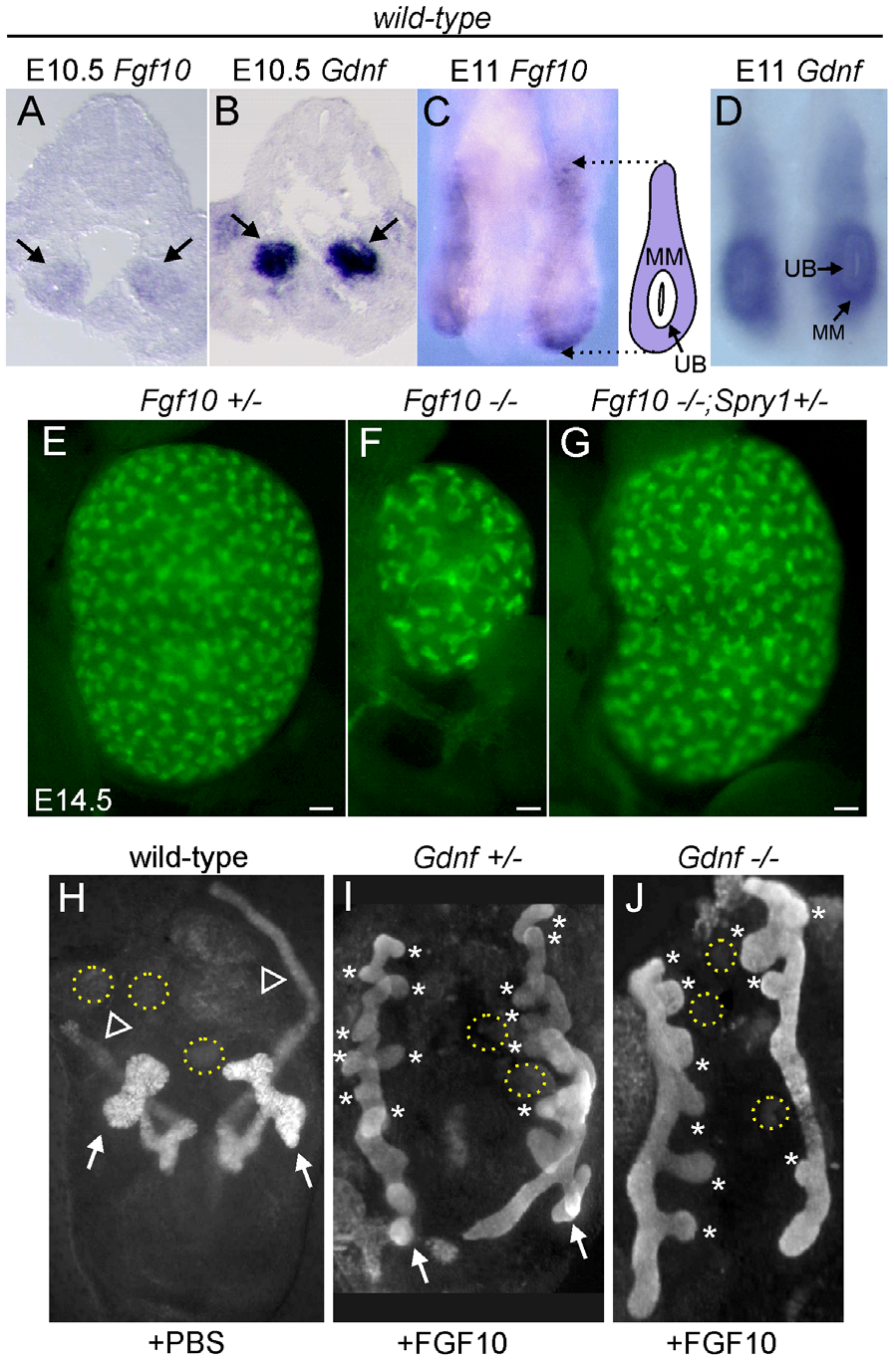
*Fgf10* expression and function in early ureter and kidney development. (A,B) *In situ* hybridization in transverse sections of E10.5 wild type embryos reveals that *Fgf10* and *Gdnf* are expressed in metanephric mesenchyme (arrows). (C,D) Whole-mount *in situ* hybridization at E11.0 (dorsal view) shows that *Fgf10* and *Gdnf* are expressed in metanephric mesenchyme (MM) surrounding the UB epithelium. The schematic diagram illustrates *Fgf10* expression, with purple indicating where the hybridization signal was detected. (E–G) Visualization of *Hoxb7/myrVenus* shows (E) normal UB branching in an *Fgf10+/−* kidney, (F) reduced branching in an *Fgf10−/−* kidney, and (G) rescue of UB branching in an *Fgf10−/−* kidney when *Spry1* dosage is reduced (*Spry1+/−*). Scale bars, 100 µm. (H–J) Induction of ectopic budding from the Wolffian duct by FGF10. Dissected E10.5 urogenital regions were cultured with control PBS-soaked beads (H) or beads soaked in FGF10 (I,J) placed between the two Wolffian ducts (dotted yellow circles). FGF10 induces multiple ectopic UB outgrowths (marked by asterisks) in both control *Gdnf+/−* (I) and *Gdnf−/−* (J) samples. Open arrowhead in H, Wolffian duct; arrows in H-I, normal ureteric buds.

We next sought to determine what signaling molecule(s) support ureteric bud outgrowth from the WD, and subsequent growth and branching, in the absence of *Gdnf/Ret* and *Spry1*. The observation that kidney development is rescued in *Gdnf−/−* or *Ret−/−* embryos only when *Spry1* is absent suggests that the signaling responsible for the rescue must itself be negatively regulated by *Spry1*. Since Sprouty genes are negative regulators of RTK signaling, the rescue most likely occurs through a RTK. According to this reasoning, FGF signaling is a strong candidate. Genetic studies in the mouse have identified FGF7 and FGF10, signaling through FGFR2, as important factors for normal UB branching [32],[33]; however, the effects of *Fgf7* or *Fgf10* knockouts (KOs) are far less severe than those caused by loss of *Gdnf* or *Ret*, indicating that these FGFs play a secondary role under normal conditions. *Fgf7* mRNA was not detected in the kidney before E14.5 [34], whereas *Fgf10*, like *Gdnf*, is expressed in the MM at least as early as E10.5 (Figure 6A–6D), making *Fgf10* a good candidate to participate in UB outgrowth and early branching morphogenesis. *Fgf10−/−* mice [35] have small kidneys at birth [33], and we found this to be reflected in reduced UB branching during kidney development (Figure 6E and 6F). The reduction in UB branching was comparable to that in kidneys lacking *Fgfr2* (or both *Fgfr1* and *Fgfr2*) in the UB lineage [36], suggesting that FGF10 is the major FGF signaling through FGFR2 in the UB. Furthermore, this defect could be corrected by deletion of one *Spry1* allele (Figure 6G), indicating that *Spry1* negatively regulates FGF10 (as well as GDNF) signaling.

**Figure 7:**
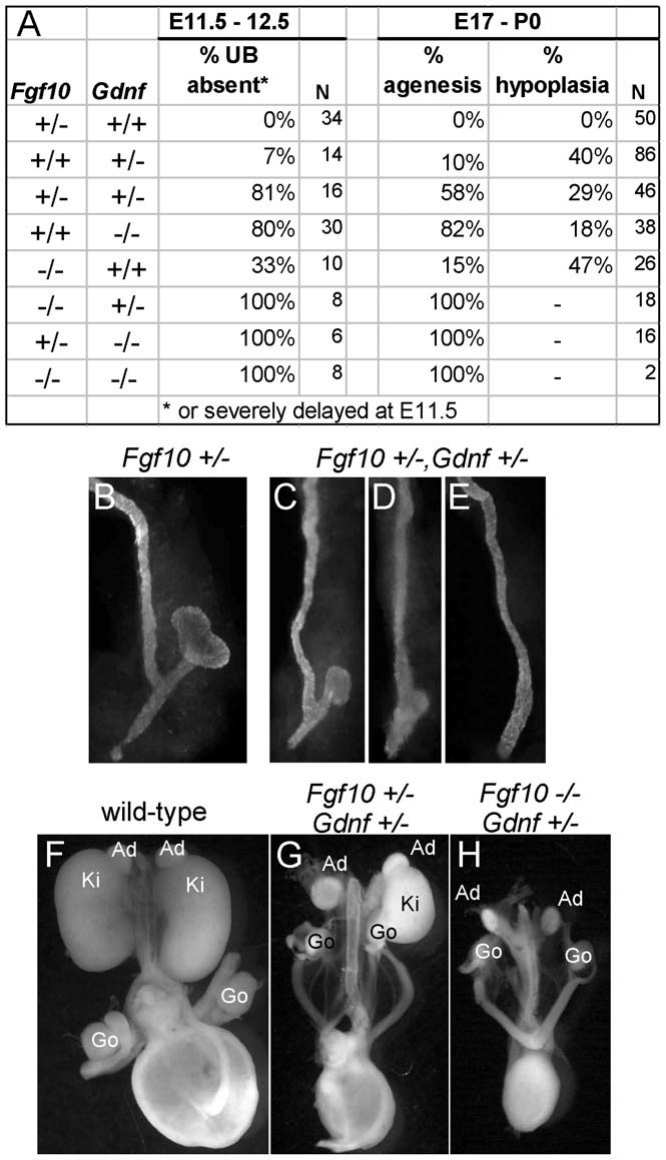
*Fgf10* and *Gdnf* cooperate to support UB outgrowth and kidney development. (A) Frequency of the failure of UB outgrowth at E11.5–12.5, and renal agenesis or hypoplasia at E17.5-P0. (B) Normal T-stage UB in an *Fgf10+/−* embryo at E11.5. (C–E) Three examples of UB formation or lack thereof in *Fgf10+/−*;*Gdnf+/−* E11.5 embryos. In (C), the UB is slightly retarded, in (D), the UB is severely delayed, and in (E) the UB is absent. (F–H), normal kidneys in wild-type and renal agenesis or hypoplasia in compound *Fgf10/Gdnf* mutants at P0. The wild-type in (F) has two normal kidneys, the double heterozygote in (G) has renal agenesis on one side and a hypoplastic kidney on the other, and the *Fgf10−/−*;*Gdnf+/−* example in (H) has bilateral agenesis. Ad, adrenal; Ki, kidney; Go, gonad. n = number of (potential) kidneys.

To examine the relationship between FGF10 and GDNF in kidney development, we performed gain- and loss-of-function studies. FGF10-soaked beads placed next to the WD of E10.5 embryos induced the formation of multiple ectopic buds (Figure 6H and 6I), as do GDNF beads [10]. To test whether FGF10 induced the ectopic buds indirectly, by up-regulating *Gdnf*, we performed the same experiment in *Gdnf−/−* embryos, but the result was similar (Figure 6J). Therefore, FGF10 is *capable* of inducing UB outgrowth, presumably by acting directly on the WD. The role of *Fgf10* was also examined by performing genetic crosses between *Fgf10* and *Gdnf* KO mice, and examining UB formation at early stages (E11.5–12.5) and kidney development in late fetal or newborn mice. *Fgf10* heterozygotes always had normal ureters and kidneys (Figure 7A and 7B), whereas *Gdnf* heterozygotes had a low frequency (7–10%) of defective UB outgrowth or renal agenesis (Figure 7A). However, in *Fgf10+/−;Gdnf+/−* double heterozygotes, 81% of the UBs were missing or severely delayed at E11.5–E12.5 (e.g., Figure 7A and 7C–7E), and 58% of kidneys were absent at E17.5–P0 (e.g., Figure 7A, 7F, and 7G), roughly equivalent to what is observed in *Gdnf* null homozygotes with normal *Fgf10* dosage (Figure 7A). Furthermore, although renal agenesis was rare in *Fgf10* homozygotes (15%), removing one *Gdnf* allele (*Fgf10−/−;Gdnf+/−*) caused 100% agenesis (e.g., Figure 7A and 7H). Thus, while the consequences of deleting both *Fgf10* alleles in a wild-type background are relatively mild, in a *Gdnf+/−* background the loss of even one *Fgf10* allele causes more severe defects, and loss of both *Fgf10* alleles is catastrophic, indicating that *Fgf10* and *Gdnf* normally cooperate to promote UB outgrowth from the WD.

**Figure 8:**
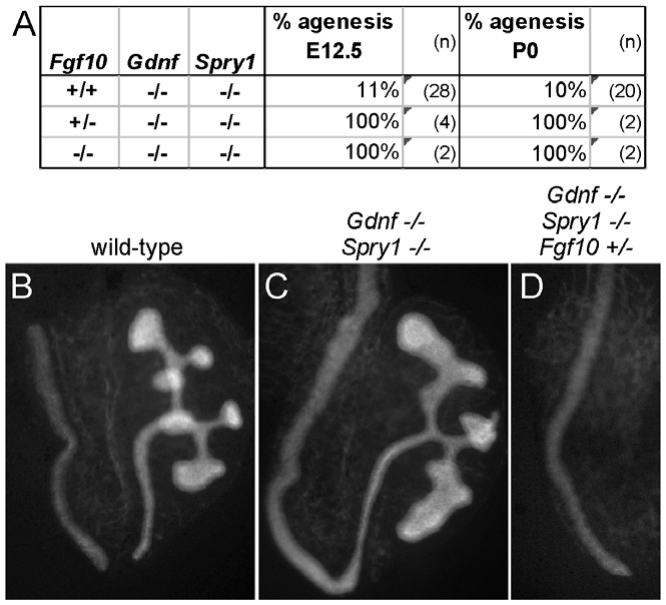
*Fgf10* is required for ureter and kidney development in the absence of *Gdnf* and *Spry1*. (A) Frequency of absence of the UB at E12.5 and renal agenesis at P0, in *Gdnf−/−*;*Spry1−/−* mice with 0, 1, or 2 *Fgf10* null alleles. (B) Example of normally branched wild-type UB at E12.5. (C) *Gdnf−/−*;*Spry1−/−* UB with moderately reduced branching at E12.5. (D) Absence of the UB in a *Gdnf−/−*;*Spry1−/−*;*Fgf10+/−* embryo at E12.5. n = number of (potential) kidneys.

To ask if it is FGF10 that rescues kidney development in *Gdnf−/−*;*Spry1−/−* mice, we next examined *Gdnf−/−;Spry1−/−* mice in which *Fgf10* gene dosage was reduced. We found that removal of either one or both *Fgf10* alleles resulted in 100% renal agenesis (Figure 8). These data conclusively demonstrate that FGF10 supports the extensive kidney development that occurs in *Gdnf−/−;Spry1−/−* mice.

**Figure 9:**
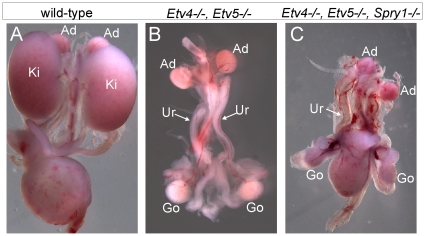
Loss of *Spry1* does not rescue kidney development in *Etv4−/−;Etv5−/−* mice. (A) Kidneys in a wild-type mouse at P0. (B) *Etv4−/−;Etv5−/−* mouse with two ureters but no kidneys. (C) *Etv4−/−;Etv5−/−;Spry1−/−* mouse with one ureter and no kidneys. Ki, kidney; Ad, adrenal gland; Ur, ureter; Go, gonad.

Expression of the ETS transcription factors ETV4 and ETV5 in the UB *in vivo* requires normal levels of GDNF/RET signaling, and they can also be upregulated in kidney cultures by exogenous FGF10, suggesting that they function downstream of both RET and FGFR2 [8]. If ETV4 and ETV5 are needed to transduce both GDNF and FGF10 signals, removing *Spry1* should be unable to rescue kidney development in *Etv4−/−;Etv5−/−* mice. In accordance with this prediction, the three triple mutant (*Etv4−/−;Etv5−/−;Spry1−/−)* mice obtained also lacked both kidneys, like *Etv4−/−;Etv5−/−* mice (Figure 9).

## Discussion

To investigate the roles of GDNF/RET signaling and negative regulation by Sprouty1 in branching morphogenesis of the Wolffian duct and ureteric bud during ureter and kidney development, we generated mice that lacked *Spry1* and either *Gdnf* or *Ret*. We found, unexpectedly, that nearly all the double homozygous mutants developed two large, well organized kidneys, with normal ureters, a highly branched collecting duct system, and extensive nephrogenesis. Thus, it appears that for many aspects of ureter and kidney development, the balance between positive signaling via GDNF/RET and negative regulation via SPRY1 is more critical than the specific role of GDNF. These observations suggested that other signaling molecules, whose activity like that of GDNF is negatively regulated by SPRY1, must be able to perform many of the functions of GDNF, but only when SPRY1 is absent. We identified FGF10 as one such factor; although knockout of *Fgf10* normally has relatively minor effects on kidney development, FGF10 plays a critical role when GDNF/RET signaling is reduced or absent. Close examination of *Gdnf−/−;Spry1−/−* and *Ret−/−;Spry1−/−* double mutant kidneys revealed that while the UB branches extensively, and proximal-distal UB patterning is retained, the characteristic branching pattern is significantly disrupted. Thus, although GDNF/RET signaling is not required for UB growth or branching *per se* (when SPRY1 is also absent), it has an apparently unique role in determining the normal branching pattern.

In a wild-type background (i.e., in the presence of SPRY1), GDNF/RET signaling is essential for the positioning and normal outgrowth of the UB from the WD. Not only does the UB usually fail to emerge in *Ret−/−* or *Gdnf−/−* mice, but when it does, its position is often abnormal, resulting in the lack of a normal connection to the bladder [37]. Furthermore, ectopic expression of *Gdnf* causes ectopic UBs to form along the WD [38]–[40]. This led to the model that the specific domain of *Gdnf* expression in the nephrogenic cord is critical for positioning the UB in the correct location [5],[16]. However, in the absence of SPRY1, mice lacking GDNF or RET make a normal UB that develops into a normal ureter connected to the bladder, as indicated by the absence of hydroureter. Therefore, signaling via another ligand/receptor that is also negatively regulated by SPRY1 must be able to properly position and induce outgrowth of the UB in the absence of GDNF or RET. We found that FGF10, presumably signaling via FGFR2, is an essential component of this alternative signaling, as removing either one or two *Fgf10* alleles in a *Gdnf−/−;Spry1−/−* background caused failure of UB emergence, leading to renal agenesis. Moreover we showed that this FGF10/FGFR2 signaling is not dependent on GDNF signaling because FGF10 induces WD budding of the cultured mouse urogenital system, even in a *Gdnf−/−* embryo. The possibility remains that other factors (other FGFs, or other signaling molecules) are also involved in this process. It has been shown that several FGFs can induce UB outgrowth from cultured rat WD, and in this assay FGF10 had relatively weak activity whereas FGF7 and other FGFs were more active [21]. The ability of FGF7 to cooperate with or replace GDNF in this process remains to be tested genetically. Other mechanisms, such as the local inhibition of BMP4 by Gremlin1 [41],[42], also contribute to the normal positioning of the UB.

Unlike *Gdnf−/−* or *Ret−/−* ureteric buds on a wild-type (*Spry1+/+*) background, which grow and branch minimally if at all, the double mutant UBs (*GGSS* or *RRSS*) grew and branched extensively, leading to a kidney that was often close to normal in size, with an extensive collecting duct system, normal overall histoarchitecture and large numbers of nephrons connected to the collecting ducts. Therefore, GDNF/RET signaling does not have a unique ability to induce UB branching, including the predominant terminal bifurcations, nor is it required for the UB tips to induce nephrogenesis. As in the case of UB outgrowth from the WD, it appears that other factors are potentially redundant with GDNF in their ability to promote UB branching. Since loss of *Fgf10* in a *Gdnf−/−;Spry1−/−* double mutant background eliminated initial UB outgrowth, it could not be determined to what extent FGF10 contributes to later UB branching in the absence of GDNF. However, the reduced UB branching in *Fgf10−/−* kidneys shows that FGF10 normally contributes significantly to UB branching, and is likely to be at least one of the factors that can promote this process in *GGSS* or *RRSS* double mutant mice. Other factors that might also be involved include HGF and EGF [43].

It was recently reported that the effects of a *Ret*-Y1062F point mutation, which causes renal agenesis or hypodysplasia similar to that observed in *Ret* knockout mice, can be rescued by removal of *Spry1* [44]. The double mutant mice had kidneys of normal size, with normal glomerular number. The Y1062F mutation abolishes signaling through the PI3K-AKT and RAS-MAPK pathways, but does not affect signaling through PLC-γ or other pathways that potentially act downstream of RET (e.g., SRC) [45]. The authors speculated that in the double mutants, the ERK MAPK pathway might be activated by RET via an alternative pathway involving PLC-γ, allowing kidney development to proceed normally, and they did not suggest that other signaling molecules might substitute for GDNF under these conditions. However, in our *Ret−/−;Spry1−/−* double null mutant mice, the ability of RET to signal through alternative pathways was eliminated, which revealed the ability of other signaling molecules, including FGF10, to support kidney development in the absence of RET or GDNF.

**Figure 10:**
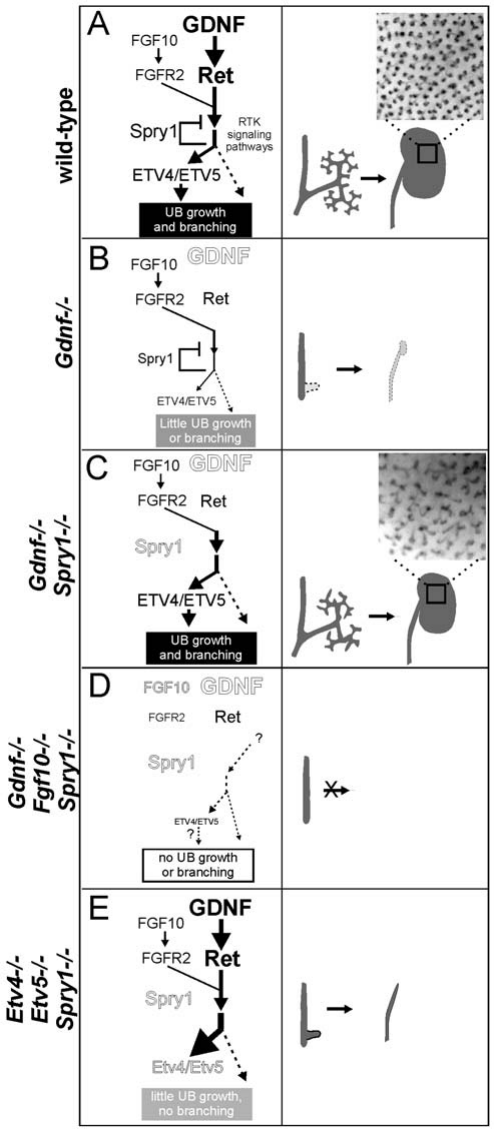
Model: GDNF and FGF10 cooperate to promote ureteric bud branching morphogenesis, via *Etv4* and *Etv5*, while Sprouty1 regulates signaling downstream of both RET and FGFR2. (A) In wild-type, GDNF/RET signaling plays a major role and FGF10/FGFR2 a minor role in promoting UB outgrowth and branching morphogenesis. The response to these signals is modulated by SPRY1, leading to a normal kidney at birth (right panel). The transcription factors ETV4 and ETV5 are downstream effectors of GDNF and FGF10 signaling. (B) In the absence of GDNF, there is presumably less SPRY1 produced [17] (indicated by smaller text), but FGF10 is insufficient to overcome negative regulation by SPRY1, causing reduced downstream signaling to induce UB budding and branching (indicated by thinner arrows), one manifestation of which is a severe reduction in *Etv4/Etv5* expression [8]. Consequently, renal agenesis or severe hypodysplasia is observed. (C) When GDNF and SPRY1 are both absent, the lack of negative regulation of signaling by FGFR2 allows for *Etv4/Etv5* expression, UB branching, and kidney development; however, the pattern of UB branching is altered, suggesting a unique role of GDNF in this process. (D) When FGF10 and GDNF are both absent, there is too little RTK signaling, even in the absence of negative regulation by SPRY1, to allow UB outgrowth from the Wolffian duct, resulting in renal agenesis (whether *Etv4/Etv5* would be expressed is not known, as there is no ureter or kidney to analyze). (E) Renal agenesis in *Etv4−/−*;*Etv5−/−* mice is not rescued by loss of *Spry1*, showing that increased RTK signaling is insufficient for kidney development in the absence of *Etv4* and *Etv5* (dashed arrow). The observation that ureters develop in *Etv4;Etv5;Spry1* triple mutants suggests that UB outgrowth, but not later branching, can occur independently of *Etv4/Etv5*. Insets in **a** and **c** show the pattern of branching UB tips in stage P0 wild-type and double mutant kidneys.

Based on our findings, we propose a model (Figure 10) in which GDNF, FGF10 and probably other signaling molecules expressed in the MM signal through their cognate receptor tyrosine kinases in the UB epithelium to collectively promote budding from the Wolffian duct and subsequent growth and branching during kidney development. RET and FGFR2 (and probably other RTKs) activate a series of shared downstream signaling pathways, including RAS-MAPK, PI3K-AKT and PLC-γ-Ca++ [46], which together support UB branching morphogenesis. *Spry1* expression is upregulated by these signals, and SPRY1 then provides negative feedback by regulating one or more of the shared signaling pathways downstream of RET and FGFR. In early kidney development, GDNF is the predominant signal, while FGF10 is much weaker (presumably due to lower expression) (Figure 10A). Expression of *Etv4* and *Etv5* is upregulated by these signals, thus controlling transcription of downstream genes required for UB growth and branching. Loss of *Gdnf* (Figure 10B) causes renal agenesis because in the presence of SPRY1 the level of FGF10 signaling via FGFR2 is not sufficient to produce the necessary responses, such as an appropriate level of *Etv4* and *Etv5* expression. Normally, loss of *Fgf10* has relatively mild consequences because of the high level of GDNF signaling. When *Spry1* is absent there is no brake on signaling via FGFR2 (Figure 10C), and GDNF can be removed without causing renal agenesis, due (at least in part) to the effects of FGF10, and to the restoration of *Etv4/Etv5* expression; however, UB branching pattern is abnormal. If *Fgf10* is also removed (Figure 10D) any remaining factors are insufficient to rescue kidney development, resulting in renal agenesis. In the absence of *Etv4* and *Etv5*, removal of *Spry1* is unable to rescue kidney development (Figure 10E). This suggests that *Etv4* and *Etv5* normally mediate the combined effects of several RTKs (RET, FGFR2 and probably others), and therefore elevated RTK signaling due to lack of SPRY1 cannot bypass the requirement for these transcription factors.

The main abnormality observed in the *GGSS* and *RRSS* double mutant kidneys was in the specific pattern of branching. Instead of the regular terminal bifurcations in wild-type kidneys, which typically occur at right angles to the previous branching event, the double mutant branching UB tips were heterogeneous in shape, spacing, orientation, branch angle and frequency of branching. The defects were distinct from those caused by loss of *Spry1* alone, which causes the UB tips to swell but does not alter branch orientation or tip spacing. Therefore, it appears that these specific defects in branching pattern are a consequence of the loss of GDNF/RET signaling, and reflect a function that cannot be replaced by FGF10 or other factors present in the double mutant kidneys.

How may GDNF/RET signaling influence the specific pattern of UB branching? One possibility is that GDNF in the metanephric mesenchyme acts as a chemoattractant to direct the growth of the UB tips toward local foci of GDNF expression [10],[14],[16], similar to the way in which FGF10 is thought to direct the branching of the developing lung epithelium [47],[48]. We have previously argued against such a model for several reasons [40]. First, the distribution of *Gdnf* mRNA in the MM is extremely diffuse; however, it remains possible that the protein is more limited in its spatial distribution than the mRNA. Second, we found that kidneys developed rather normally in *Gdnf* null mice in which *Gdnf* was misexpressed in the UB epithelium, suggesting that it is the presence, but not the location, of GDNF that is important [40]. However, the specific pattern of UB branching was not closely examined in those mutant/transgenic mice, and it remains possible that they had subtle branching defects similar to the *GGSS* and *RRSS* double mutant kidneys. Methods to locally and precisely manipulate the pattern of *Gdnf* expression will be needed to better test this model. If not through chemoattraction, then GDNF/RET signaling must in some other manner influence the specific pattern of growth and branching of the UB tips.

## Methods

### Ethics statement

All work on animals was conducted under PHS guidelines and approved by the relevant Institutional Animal Care and Use Committees.

### Mouse strains

*Ret* [4], *Gdnf* [3], *Spry1* [17], *Fgf10* [35], *Etv4* [49], *Etv5* [50] and *HoxB7/myrVenus* [27] mutant mice have been described. These mice were maintained on a mixed background (129S1/SvmJ:C57BL/6). Embryo stage was estimated by considering noon of the day of the vaginal plug as embryonic day (E) 0.5, and more accurate staging was determined by counting somites. PCR genotyping of mice and embryos was done as described previously [3],[4],[8],[17],[35].

### Whole-mount *in situ* hybridization

Whole-mount and section RNA *in situ* hybridization and detection of β-galactosidase activity were performed as described previously [38],[51] using digoxigenin-UTP-labeled anti-sense riboprobes.

### Histological analysis and nephron counting

Newborn mice were sacrificed according to Institutional and NIH guidelines. Whole kidneys and urogenital tracts were dissected in PBS. Kidney cross-sectional area was determined from whole-mount photographs of 28 wt, 16 *GGSS* and 12 *RRSS* P0 kidneys using ImageJ. For histological analysis, 7–10 µm sections were prepared from paraffin-embedded samples fixed in 4% paraformaldehyde (PFA). De-waxed sections were stained with either haematoxylin and eosin (H&E) or Periodic Acid Schiff (PAS). To count glomeruli, five evenly-spaced sections across each kidney (two wild-type and four *RRSS* mutants) were stained with podocalyxin, and the number of glomeruli per section was averaged for each kidney.

### Metanephric kidney explant cultures and immunohistochemistry

Intermediate mesoderm or metanephric kidneys were dissected from E10.5 to E14.5 embryos in PBS +Ca +Mg (Invitrogen). Explants were cultured at 37°C in DMEM/F12 (Invitrogen) supplemented with Glutamax, 100 U/ml penicillin, 100 µg/ml streptomycin, and 10% fetal bovine serum in a 5% CO_2_, humidified atmosphere at the medium-air interface on Costar Transwell filters (0.4 µm). After culture, explants were fixed in 4% PFA. For immunostaining, explants were incubated with goat anti-podocalyxin antibodies (R&D Systems), followed by Cy2 or Cy3 anti-goat Ig (Jackson ImmunoResearch). Images were captured on a Zeiss Axio Observer Z1.

For FGF bead experiments, posterior intermediate mesoderm was dissected from embryos at the 29 to 35 somite stage in Hanks Balanced Salt Solution, with 1% FBS. Two heparin acrylic beads (Sigma) soaked in FGF10 (R&D Systems), reconstituted in PBS at 1mg/ml) or in PBS were inserted between the WDs, and rudiments were cultured on Whatman Nuclepore Track-Etch Membrane filters (8 micron pore size) in 44% F12, 44% DMEM, 10% FBS, 1% glutamine, 1% Penstrep at the air-liquid interphase for 48–55 hours. Samples were fixed in cold 100% methanol and stained with anti-pan cytokeratin antibody (Sigma C9687).

### Confocal imaging and 3D analysis

E15.5 metanephric kidneys were dissected in PBS and fixed overnight in 4% PFA. After clearing using FocusClear (CelExplorer), kidneys were mounted in MountClear (CelExplorer) and scanned using a Leica LS5 confocal microscope, and 3D rendering was performed using Volocity software.
